# Multiobjective Optimization Design of Spinal Pedicle Screws Using Neural Networks and Genetic Algorithm: Mathematical Models and Mechanical Validation

**DOI:** 10.1155/2013/462875

**Published:** 2013-07-31

**Authors:** Yongyut Amaritsakul, Ching-Kong Chao, Jinn Lin

**Affiliations:** ^1^Department of Mechanical Engineering, National Taiwan University of Science and Technology 43, Section 4, Keelung Road, Taipei 106, Taiwan; ^2^Department of Orthopaedic Surgery, National Taiwan University Hospital 7, Chung-Shun South Road, Taipei 100, Taiwan

## Abstract

Short-segment instrumentation for spine fractures is threatened by *relatively high* failure rates. Failure of the spinal pedicle screws including breakage and loosening may jeopardize the fixation integrity and lead to treatment failure. Two important design objectives, bending strength and pullout strength, may conflict with each other and warrant a multiobjective optimization study. In the present study using the three-dimensional finite element (FE) analytical results based on an L_25_ orthogonal array, bending and pullout objective functions were developed by an artificial neural network (ANN) algorithm, and the trade-off solutions known as Pareto optima were explored by a genetic algorithm (GA). The results showed that the knee solutions of the Pareto fronts with both high bending and pullout strength ranged from 92% to 94% of their maxima, respectively. In mechanical validation, the results of mathematical analyses were closely related to those of experimental tests with a correlation coefficient of −0.91 for bending and 0.93 for pullout (*P* < 0.01 for both). The optimal design had significantly higher fatigue life (*P* < 0.01) and comparable pullout strength as compared with commercial screws. Multiobjective optimization study of spinal pedicle screws using the hybrid of ANN and GA could achieve an ideal with high bending and pullout performances simultaneously.

## 1. Introduction

 The treatment goals of spinal fractures include deformity correction, neurological decompression, and fixation of the instability [1]. Transpedicle screw fixators can achieve reduction, decompression, and fixation at the same time, and provide high fixation stability for early mobilization [2–4]. One important principle of spinal fixation is to minimize the instrumentation levels to reduce the surgical trauma, preserve the motion segments, and avoid junctional arthritis and late back pain caused by long-segment instrumentation which may increase load at the adjacent segments [5]. However, *relatively high* failure rates with this short-segment instrumentation which fixes only one level above and below the fractured vertebra have been reported [1]. Failure of the pedicle screws including breakage and loosening may jeopardize the fixation integrity and lead to treatment failure [6–8]. Especially, broken pedicle screws trapped in the vertebral bodies are difficult to retrieve and may interfere with subsequent revision surgeries [9]. Thus, the design rationale of pedicle screws is to increase bending strength to resist breakage and pullout strength to resist loosening simultaneously [10–12]. However, these two design objectives are closely related to the screws' structures and may conflict with each other [10, 13–15]. Improving one objective may cause deterioration of the other. Therefore, optimization studies to improve both design objectives simultaneously are critical but still *rare* in the literature [16].

In the present study, computer aided engineering (CAE) with high calculation technology was applied. The pedicle screws were analyzed with finite element (FE) models, and then artificial neural network (ANN) algorithms were adopted to model the analytical process. The optimal screw design was achieved via an evolutionary multiobjective approach with a genetic algorithm (GA) [15]. Last, mechanical tests were conducted to validate the optimal design by comparison with commercially available devices.

## 2. Materials and Methods

### 2.1. Screw Structures and Orthogonal Array of Taguchi Robust Design Methods [15]

**Figure 1:**
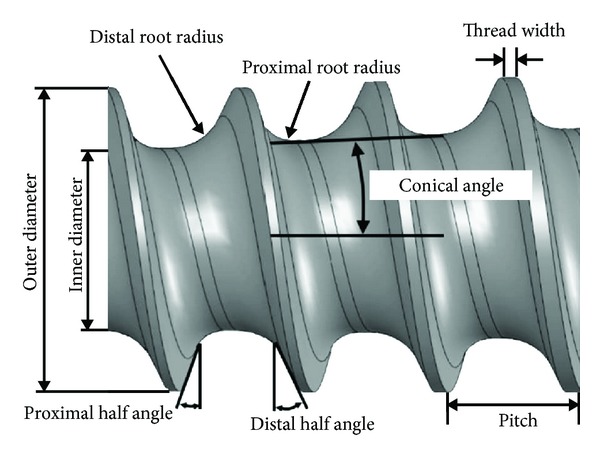
Illustration of structural variables for the spinal pedicle screw.

In this study the outer diameter of the pedicle screws was fixed at 7 mm. Among the eight independent structural variables of pedicle screws, six of them were analyzed including beginning position of conical angle (BP), inner diameter (ID) at the screw tip, proximal root radius (PRR), pitch (P), proximal half angle (PHA), and thread width (TW) (Figure 1). The design space of these structural variables was determined according to commonly used pedicle screws and previous studies [1, 15]: 0–36 mm for BP, 3.8–5.5 mm for ID, 0.4–1.0 mm for PRR, 2.6–4.0 mm for P, 5–20° for PHA, and 0.1–0.3 mm for TW (Table 1). The distal root radius and distal half angle were fixed at 1 mm and 25°, respectively, because of minimal effects on the mechanical performance of the screws [17, 18]. An L_25_ orthogonal array for six factors with five levels was selected for optimization study. This orthogonal array ensures a balanced comparison of levels of each structural variable and represents the entire experimental space. The structural variables were equally divided to 5 levels and put in the L_25_ orthogonal array. All the screw designs in the orthogonal array fulfilled the geometric constraints [15].

### 2.2. FE Models

**Figure 2:**
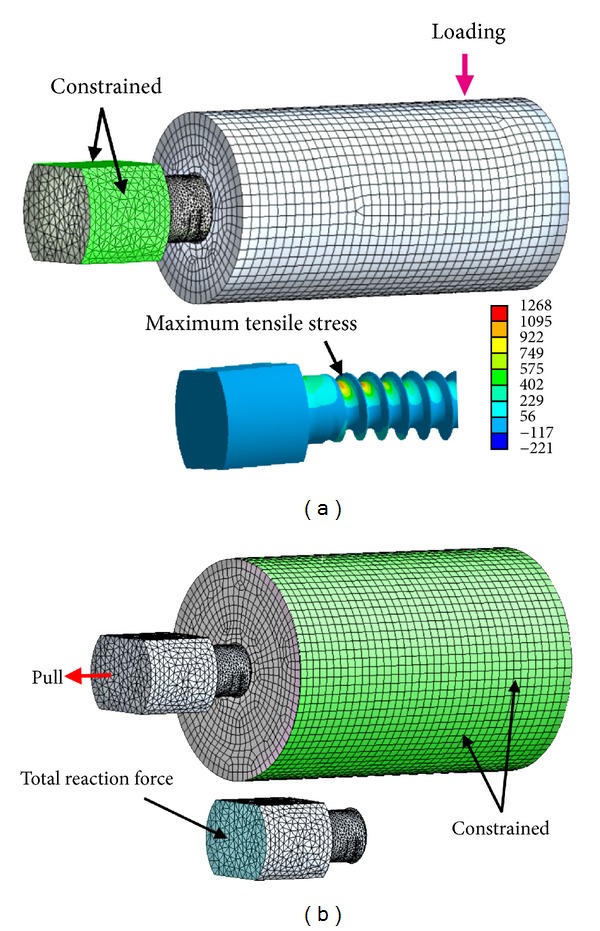
Finite element models: bending (a) and pullout (b).

Three-dimensional solid models of the spinal pedicle screws inserted at the center of a cylinder were first created by the CAD software SolidWorks 2005 (SolidWorks, Concord, MA, USA) and then imported into the CAE software ANSYS 10 Workbench (ANSYS Inc., Canonsburg, PA, USA) with the use of the Parasolid format (Figure 2). The screw was 45 mm, and the cylinder was 60 mm in length. The pedicle screw was free-meshed with high order 10-node tetrahedral elements, and the cylinder was map-meshed with 20-node hexahedral elements with the element size of 1.2 mm. Surface-to-surface contact elements were used for the interface between the pedicle screw and cylinder with a frictionless condition. No axial rotation of the constructs was allowed. The elastic modulus of titanium pedicle screws was 114 GPa. The Poisson's ratio was 0.3 for both pedicle screw and cylinder. The thread valleys with stress concentration were remeshed, and the numerical convergence was confirmed by increasing mesh density. 

For bending strength, a cantilever bending setup was used to simulate the worst-case scenario of total corpectomy conditions. The cylinders with an outer diameter of 20 mm were made from homogeneous polyoxymethylene with an elastic modulus of 2.6 GPa. The screw head was constrained, and a compressive force of 225 N was applied to the cylinder with a lever arm of 40 mm (Figure 2(a)). In the postprocessing, the maximum tensile stress of the pedicle screw was recorded to represent the bending strength. Lower maximum tensile stress represented longer fatigue life and higher bending strength, and vice versa. For pullout strength, to simulate the worst case scenario of osteoporosis, the cylinder with an outer diameter of 30 mm was assumed to be osteoporotic bone with an elastic modulus of 137.5 MPa. The effects of bone compaction caused by conical screws were simulated by adjusting the elastic modulus of the bone surrounding the conical core according to the density change of the surrounding bone [11]. Density change was calculated on the basis of the volume reduction caused by the compaction. The elastic modulus of bone was assumed to be a power-law function of the density with an exponent of 2. In the loading condition, an axial displacement of 0.01 mm was applied to the end surface of the pedicle screw. The boundary conditions were constraints at the outer surface of the cylinder (Figure 2(b)). In the postprocessing, total reaction force on screws, defined as the summation of the resultant axial force at the surface of the screw, was recorded. Higher total reaction force represented stronger pullout strength. 

### 2.3. Artificial Neural Network Modeling

**Figure 3:**
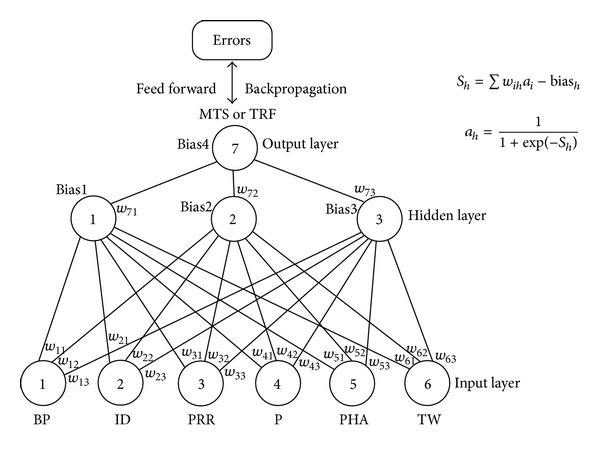
Three-layer feed-forward error—backpropagation neural network model.

ANN as a regression device containing layers of computing nodes with remarkable information processing capability can detect nonlinearities by machine learning and adaptability based on the least- squares algorithm [19]. In the current study, because of the complexity of FE analyses, ANN was used to replace the FE models of bending strength and pullout strength for construction of the objective functions for multiobjective optimization studies. The supervised feed-forward error-backpropagation learning models with sigmoid activation function were developed. Six structural variables were used as inputs, and single output was either maximum tensile stress or total reaction force (Figure 3). A three-layered ANN based on the 25 screw designs in the orthogonal array with three neurons in one hidden layer was used as the learning set. Another testing set with 10 randomly selected screw designs outside the orthogonal array was used to supervise the learning process. The input quantities were normalized to a range from −1 to 1, and the output quantities were normalized to a range from 0 to 1. The initial weights and the biases between −1 to 1 were randomly assigned. Both learning rate and the coefficient of momentum term were set at 0.5. The new weight and bias were updated as the error between the predicted and the target performance was minimized. Generally, the learning and testing errors kept decreasing during computing iterations. The process was terminated when the testing errors were minimal. The ANN models were run 100 times with different initial weights, and the best model with the least test error was selected for optimization study. The ANN is coded by Microsoft Visual Basic (Redmond, WA).

### 2.4. Multiobjective Optimization with GAs

**Figure 4:**
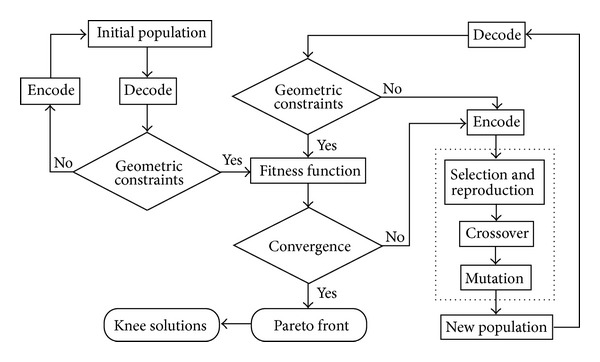
Flowchart of evolutionary optimality in GAs.

GA is commonly used for multiobjective optimization by using stochastic operators (Figure 4). The biobjective problem of the screw functions could be expressed by an aggregated weighted-sum fitness function (*F*): *F* = *w* · *F*_bending_ + (1 − *w*) · *F*_pullout_, where *F*_bending_ was the normalized objective function of bending; *F*_pullout_ was the normalized objective function of pullout; *w*, the given weight, was systematically changed from 0 to 1 to assess the different combinations of both performances. Both objective functions were transformed into the-larger-the-better problem before aggregation, and the fitness function (*F*) was maximized. The algorithm was initiated with a population with 40 randomly selected chromosomes. Each chromosome was composed of six design parameters with 42 bits of zeros and ones. The optimization process included selection, reproduction, and termination. Roulette wheel selection replicates the fitter solutions found in the population. Then a second generation population was reproduced from those selected through genetic operators: *crossover* and *mutation*. The crossover rate and the mutation rate were 90% and 1%, respectively. If the new generations fulfilled the constraints, the fitness of the new populations was calculated and reselected again. The process was repeated and terminated until the highest ranking solution's fitness converged. The program of GAs was also developed by Microsoft Visual Basic. The optimization strategy produced a set of Pareto front with nondominant solutions, which meant there were no solutions better than those in both objectives. The optimal design range at the knee region of the Pareto front was subjectively defined as a less than 2% difference between the normalized objectives. The knee solutions were validated with FE analyses and compared with the commercially available pedicle screws. Ten thousand randomly selected screw designs were used to validate the Pareto set obtained in GA.

### 2.5. Mechanical Validation Tests

**Figure 5:**
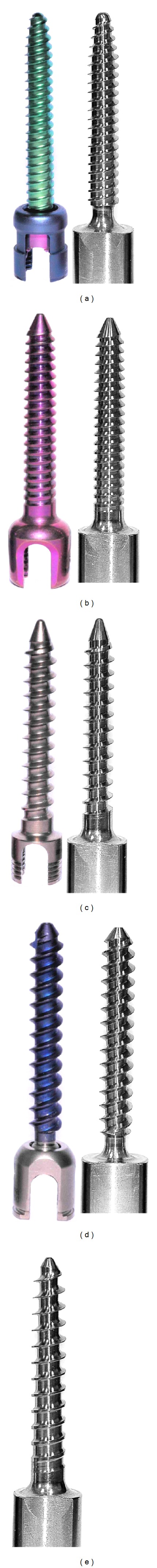
Tested pedicle screws: (a) Synthes, (b) A-Spine, (c) Moss Miami, (d) Viper, and (e) Optimal.

The results in the mathematical studies were validated by mechanical tests as conducted in the literature [1]. One optimal design randomly selected from the knee region of the Pareto front was compared with the four commercially available pedicle screws with a 7 mm outer diameter in both bending and pullout tests: Synthes (Synthes, Paoli, PA, USA), A-Spine (A-Spine Asia, Taipei, Taiwan), Moss Miami, and Viper (DePuy Spine, Raynham, MA, USA) (Figure 5). The structures of the commercial screws were measured by measuring microscope (Meiji MC-50T, New York Microscope, Hicksville, NY). To make the comparison fair, the screws were manufactured with the same titanium alloy by the same process. The mechanical tests were conducted on a materials testing machine (Bionix 858, MTS Corporation, Minneapolis, MN, USA), and the testing setup was similar to that in the FE models. In bending, polyoxymethylene cylinders (Universal Plastics, Auckland, New Zealand) representing the vertebrae could eliminate the interspecimen variability and prevent specimen failure during experiments. Sinusoidal waveform cyclic loading fatigue tests with a frequency of 10 Hz were performed with screws submerged in a saline bath at 37°C. The maximal load of the cyclic testing was 410 N with a stress ratio of 10%. The tests were terminated when the screws cracked or the number of testing cycles was more than one million [6]. The number of cycles at failure was recorded. For pullout, cellular polyurethane foam (Pacific Research Laboratories, Vashon, WA, USA) conforming to the standard of ASTM F1839-97 [20] can prevent the widely varying testing results. Two densities of the foam—0.32 and 0.16 gm/cm^3^ with a compressive modulus of 137.5 and 23 MPa and a porosity of 71% and 86%, respectively—were used to simulate cancellous bones with osteoporosis. For a fair comparison, the predrill hole was the same size as that of the ID of each screw at the screw tip. Thus, the conical screws could generate bone compaction during screw insertion. The screws were freely extracted in longitudinal direction with a loading rate of 5 mm/minute. The maximum load was defined as the pullout strength.

## 3. Results

In FE analyses, total element number ranged from 122,550 to 189,224 for bending and from 142,066 to 278,211 for pullout. The maximum tensile stress in bending tests was located at the proximal threads near the screw hub. The pedicle screws in pullout tests had negligible deformation because the bone was assumed osteoporotic (Figure 2). These two findings were compatible with the results in the mechanical tests. In ANN analyses, the computing iteration was 10000 cycles for bending and 5000 cycles for pullout. The differences between prophetic outputs obtained in ANN models and FE results were minimal. For bending, the mean absolute error was 1% (0.05~3%) for learning and 1.64% (0.03~4.13%) for testing. For pullout, the mean absolute error was 0.4% (0.03~0.88%) for learning and 0.78% (0.08~2.51%) for testing. 

**Figure 6:**
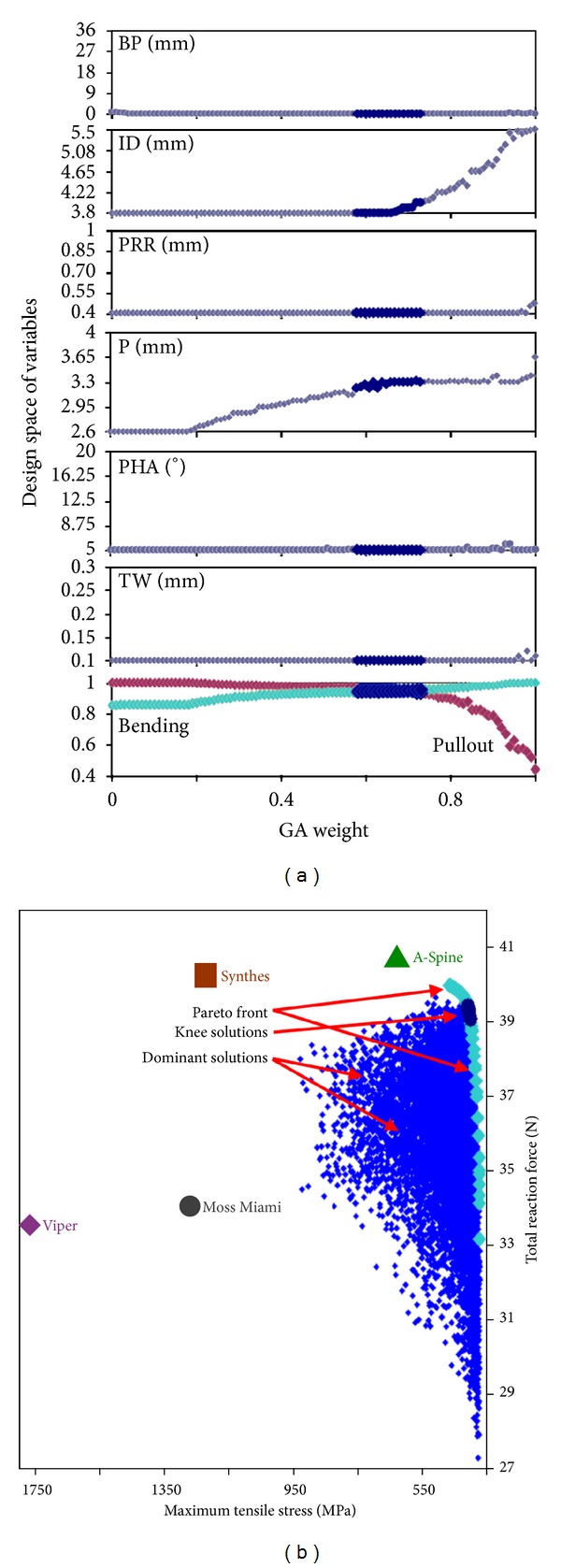
Optimization results: (a) changes of the values of structural variables related to the normalized objective functions of bending and pullout corresponding to given weights in GA. (b) Pareto plot with knee solutions.

The solutions of GA converged after 300 generations (see Supplementary Materials available at http://dx.doi.org/10.1155/2013/462875). The main factors that affected the Pareto set were ID and pitch (Figure 6), which increased along with the weight (*w*). In the knee region, the weight ranged from 0.60 to 0.72. The corresponding range of the structural variables was 3.8 to 4.06 mm for ID and 3.21 to 3.3 mm for pitch; the fixed variables were 0 mm for BP, 0.4 mm for PRR, 5° for PHA, and 0.1 mm for TW. The bending strength and the pullout strength ranged between 92% and 94% of their maxima. The exactitude of knee solutions closely approximated the results of FE analyses. The ten thousand randomly selected screw designs were all dominant solutions in Pareto plot. The commercially available pedicle screws were far away from the knee solutions. The A-Spine and Synthes type screws had high pullout strength but relatively low bending strength. Moss Miami and Viper type screws were low in both bending strength and pullout strength.

In the mechanical tests, the logarithm of the fatigue life was closely related to the maximum tensile stress obtained in FE analyses with a correlation coefficient of −0.91 (*P* < 0.01), and the pullout strength was closely related to the total reaction force with a correlation coefficient of 0.93 (*P* < 0.01) (Table 2). The optimal designs had significantly higher fatigue lives (>10^6^ cycles) than all the commercial screws by an analysis of variance test (*P* < 0.01, resp.), and pullout strength was higher than Moss Miami and Viper screws (*P* < 0.01 for both foam densities). Synthes and A-Spine screws had higher pullout strength than optimal designs, but the bending strength was relatively low because of a very small pitch (2 mm). This was compatible with the findings in FE analyses. 

## 4. Discussion

In order to reduce the incidence of fixation failure in short-segment fixation for spinal fractures, different kinds of interventions have been developed, including combined anterior instrumentation [21], bone cement augmentation [22], transpedicular vertebroplasty [23], and so forth. However, these methods are threatened by complications [1]. Improvement of the pedicle screw design to achieve better bending strength and bone holding power is still the most basic step to prevent failure of fixation. Investigating only one mechanical performance of bending strength or pullout strength of the pedicle screws exclusively might lead to undetected compromise of the other one, because these two objectives would conflict with each other in the design process [1, 15]. In the present study, with adequate control of the design space, the two mechanical performances of the screws were investigated simultaneously with ANN and GA for multiobjective optimization analysis.

FE analysis, a powerful tool for biomechanical researches on structures with complicated loading and boundary conditions [24, 25], can be reliably used for predicting the bending strength and pullout strength of orthopedic screws [17, 18]. In the present study, the FE models could be well validated by mechanical tests in both bending and pullout tests with very high correlation coefficients. However, because of the sophisticated computation process, FE analyses are not suitable for multiobjective design optimization studies. Therefore, the ANN algorithms, which have the special advantage of functional approximation with fast computation, can be used as surrogate functions of FE models for multiobjective optimization studies.

ANN, a nonlinear statistical data modeling tool, uses learning rules to develop models and parallel computing to find answers. These neurocomputing procedures mimic information processing and knowledge acquisition in human brains. ANN can construct complex relationships between input variables and output performances and process not only values but also texts, images, and voices [19, 26]. Its attractiveness comes from the remarkable information processing characteristics of the biological systems such as nonlinearity with better fit to the data, high parallelism, robustness, fault tolerance, learning, ability to handle imprecise and fuzzy information, and their capability to generalize. Our previous optimization study of tibial locking screws developed objective functions with least-squares linear regression models [15]. However, with more complex trends in the conical core design of pedicle screws in the present study, linear regression analysis with high order polynomials might fit badly at the extreme of the independent variables or in data with limiting behaviors, because polynomials do not have asymptotics [27]. ANN viewed as generalizations of “super regression” can outperform statistical regression with regard to prediction accuracy. This superiority increases as the dimensionality and/or nonlinearity of the problem increases. Classically, development of an ANN requires partitioning of the parent database. This may decrease the statistical power. In the present study, use of all the datasets in the orthogonal array in the training and 10 testing datasets randomly selected from the entire parametric space outside the orthogonal array could avoid this disadvantage and increase the predictability. 

Many real-world problems involve multiple competing objectives. The two objectives of pedicle screws, bending strength and pullout strength, are conflicting and characterized by the fact that improving one objective may jeopardize the other [15]. The present multiobjective optimization study used a weighted-sum function and GA to develop Pareto optima that were trade-off solutions for the conflicting objectives [28]. The solutions at the knee region of the Pareto front, characterized by the fact that a small improvement in either objective might cause a substantial change in the other, were considered the most suitable trade-offs (the optimal designs) by designers. The bending strength and the pullout strength of the optimal designs ranged between 92% and 94% of their maxima. This indicated that with minimal compromise of one objective, the other still could maintain a relatively high performance. However, this multiobjective optimization principle is not adequately considered in the design of commercially available pedicle screws. The Synthes and A-Spine type screws with a very small pitch (2 mm) had very high pullout strength, but such a small pitch led to a sharp root radius and high tensile stress. A small increase of maximum tensile stress might markedly decrease the fatigue life because of the logarithmic relationship. This was the reason why small pitch was not included in the design space of the present study. In contrast, both Viper and Moss Miami type screws with a cylindrical core had low bending and pullout strength. They were dominated designs, very far away from the knee region. Basically, tapering of the ID from the screw tip all the way to the screw hub may increase the bending strength and pullout strength simultaneously. Especially, elimination of the step-off at the screw hub can increase the fatigue strength substantially [1]. This explained higher fatigue life in A-Spine type and optimal design screws. Viper type screws with a smaller core at the screw hub for better adjustability of the polyaxial design might jeopardize the bending strength extremely. 

The present study has potential pitfalls. First, ANN is an empirical model and its success depends on both the quality and quantity of the data. Although only 25 datasets were used for training, the ANN model still could accurately reflect the FE results, because the orthogonal array could fairly represent the entire parametric space and the FE data were relatively noise free, as compared with clinical data. Second, a different outer diameter and range of design space might affect the ranges of the optimal design. The present study considered only screws with an outer diameter of 7.0 mm, but the design space could cover the important ranges of the pedicle screw design. Third, GAs are stochastic iterative processes and do not guarantee a global optimality. However, the optimal designs in the present study with fitness levels up to 92% or 94% of their maxima were already practically acceptable. Fourth, the ANN is criticized as a “black box” method. One cannot exactly explain what interactions are modeled in the hidden layers, and there is still no specific method to define the optimal hidden layers. However, these did not affect the method's robustness in the present optimization study. Last, the optimal design was closely related to the relative weight between the bending strength and pullout strength (1 : 1 in the present study). The selection depended on the factors linked to the problem and a thorough knowledge of them. 

In conclusion, the ANN model could reliably approximate the results of sophisticated mathematical analyses of pedicle screws. The model could be used to solve the problems of conflicting objectives of pedicle screws with evolutionary GA. The trade-off optimal solutions obtained in this optimization study could achieve an ideal with high performance in both bending and pullout tests. The present method proves beneficial to both manufacturers who design implants and surgeons who select the best product to prevent failure in the treatment of spine fractures. 

## Supplementary Material

Multiobjective optimization process using genetic algorism.

## Figures and Tables

**Table 1:** Design variables of the pedicle screws, FE analytical results and ANN models.

No.	BP (mm)	ID (mm)	PRR (mm)	P (mm)	PHA (°)	TW (mm)	MTS-FE (MPa)	MTS-ANN (MPa)	TRF-FE (N)	TRF-ANN (N)
1	0	3.80	0.40	2.60	5.00	0.10	464.49	464.71	39.93	39.99
2	0	4.23	0.55	2.95	8.75	0.15	416.30	415.05	38.40	38.59
3	0	4.65	0.70	3.30	12.50	0.20	399.83	392.63	35.56	35.75
4	0	5.08	0.85	3.65	16.25	0.25	377.19	382.49	31.37	31.26
5	0	5.50	1.00	4.00	20.00	0.30	380.56	377.39	26.36	26.48
6	9	3.80	0.55	3.30	16.25	0.30	452.25	457.14	38.37	38.38
7	9	4.23	0.70	3.65	20.00	0.10	415.59	419.10	36.48	36.57
8	9	4.65	0.85	4.00	5.00	0.15	404.94	405.94	33.66	33.72
9	9	5.08	1.00	2.60	8.75	0.20	432.04	427.96	33.96	33.82
10	9	5.50	0.40	2.95	12.50	0.25	393.19	404.48	35.67	35.54
11	18	3.80	0.70	4.00	8.75	0.25	468.89	468.30	36.74	36.80
12	18	4.23	0.85	2.60	12.50	0.30	576.54	574.13	37.94	37.90
13	18	4.65	1.00	2.95	16.25	0.10	444.50	437.91	35.72	35.71
14	18	5.08	0.40	3.30	20.00	0.15	429.80	416.79	36.65	36.33
15	18	5.50	0.55	3.65	5.00	0.20	391.64	397.72	33.37	33.49
16	27	3.80	0.85	2.95	20.00	0.20	608.56	617.34	37.47	37.15
17	27	4.23	1.00	3.30	5.00	0.25	532.07	529.34	36.37	36.23
18	27	4.65	0.40	3.65	8.75	0.30	491.02	481.84	36.73	36.58
19	27	5.08	0.55	4.00	12.50	0.10	460.59	463.83	34.67	34.41
20	27	5.50	0.70	2.60	16.25	0.15	463.35	469.46	35.14	35.44
21	36	3.80	1.00	3.65	12.50	0.15	919.85	918.26	35.19	35.31
22	36	4.23	0.40	4.00	16.25	0.20	787.66	789.04	34.90	35.05
23	36	4.65	0.55	2.60	20.00	0.25	770.40	765.61	36.66	36.90
24	36	5.08	0.70	2.95	5.00	0.30	605.92	611.19	35.97	36.01
25	36	5.50	0.85	3.30	8.75	0.10	527.13	526.30	33.54	33.46
26	25.4	4.7	0.748	3.1	9.53	0.255	488.62	483.69	36.59	36.41
27	0.5	5.093	0.885	3.59	5.68	0.183	375.29	380.79	31.47	31.64
28	31.05	5.144	0.624	3.947	18.07	0.11	504.19	510.02	34.01	33.62
29	16.87	4.3	0.774	3.507	8.95	0.156	444.71	436.37	36.46	36.73
30	25.02	5.46	0.546	3.347	6.58	0.3	429.47	427.33	34.48	34.43
31	10.24	3.87	0.577	3.135	9.515	0.28	447.07	465.53	38.51	38.73
32	6.696	4.79	0.448	3.241	18.58	0.152	412.10	401.96	37.189	37.16
33	23.25	4.39	0.463	2.86	6.16	0.187	495.95	506.34	38.365	38.56
34	21.55	5.334	0.745	2.943	17.91	0.115	420.82	424.14	34.036	34.89
35	31.64	5.49	0.543	3.528	14.39	0.294	476.86	472.29	34.167	33.80

No. 1–25, learning set; No. 26–35, testing set. MTS represents maximum tensile stress. TRF represents total reaction force.

**Table 2:** Structures and results of FE analyses and mechanical tests of four commercially available pedicle screws and the optimal design. Values were expressed as mean (standard deviation).

Mechanical properties	Synthes type	A-Spine type	Moss Miami type	Viper type	Optimal design
BP (mm)	0	0	40	cylindrical	0
CD (mm)	2.76	4	4.61	4.4	3.8
PRR (mm)	0.22	0.1	3	3	0.4
P (mm)	2	2	2.95	2.87	3.3
PHA (°)	0	0	31.35	29.93	5
TW (mm)	0.1	0.1	0.2	0.33	0.1
Maximum tensile stress (MPa)	1220	628	1268	1766	422
Total reaction force (N)	40.25	40.77	33.53	34.04	39.1
Fatigue life (10^3^ cycles)	13.77 (4.62)	46.53 (15.9)	8.52 (1.35)	—	>1000
Pullout strength, 0.32 g/cm^3^ (N)	2148 (144)	2068 (117)	1598 (56)	1553 (84)	2009 (74)
Pullout strength, 0.16 g/cm^3^ (N)	1015 (74)	951 (48)	705 (48)	662 (63)	825 (52)

Cyclic loading tests of Viper type screws were not completed because the screws yielded quickly during the tests.
